# Simulating the Hubbard model with moiré semiconductors

**DOI:** 10.1093/nsr/nwag069

**Published:** 2026-02-02

**Authors:** Kin Fai Mak, Jie Shan

**Affiliations:** Max Planck Institute for the Structure and Dynamics of Matter, Germany; School of Applied and Engineering Physics, Cornell University, USA; Laboratory of Atomic and Solid State Physics, Cornell University, USA; Kavli Institute at Cornell for Nanoscale Science, USA; Max Planck Institute for the Structure and Dynamics of Matter, Germany; School of Applied and Engineering Physics, Cornell University, USA; Laboratory of Atomic and Solid State Physics, Cornell University, USA; Kavli Institute at Cornell for Nanoscale Science, USA

## Abstract

Moiré semiconductors provide a solid-state Hubbard-model simulator, enabling triangular/honeycomb lattice realizations and tunable phase diagrams via the interaction-to-bandwidth ratio ??/??

The Hubbard model is a simple theoretical model attempting to describe the physics of interacting quantum particles in a lattice [1]. The Hubbard Hamiltonian consists of two terms: a nearest-neighbor hopping (*t*) that drives delocalization and wave-like behavior and an onsite Coulomb repulsion (*U*) that promotes localization and particle-like behavior of the quantum particles. The model is believed to capture the essential physics of many strongly correlated phenomena, including high-temperature superconductivity [2], magnetism [1], metal–insulator transition [3] and intertwined orders [4]. Although the Hamiltonian appears simple, it remains difficult to solve accurately except for the 1D cases [5]. Physical realization of the Hubbard model in higher dimensions is, therefore, highly desirable: phases of matter and their dynamics can be simulated as a function of tuning parameters like *t, U*, temperature (*T*), chemical potential (*μ*), and magnetic field (*B*). While tremendous progress has already been made with cold-atom Hubbard model simulators [6], the past several years have witnessed significant developments in a new class of Hubbard model simulators based on transition metal dichalcogenide (TMD) moiré semiconductors. In this perspective, we will briefly discuss the major developments and future opportunities in this research field.

## ANGLE-ALIGNED TMD HETEROBILAYERS

**Figure 1.**
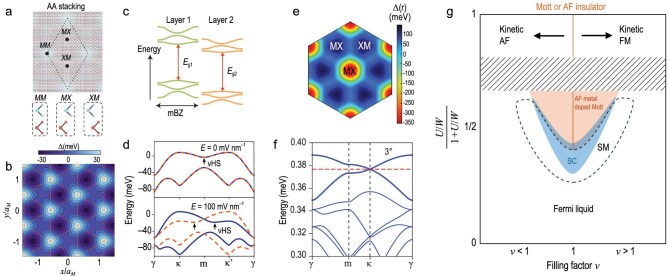
Moiré semiconductors as a Hubbard model simulator. (a) AA-stacked TMD semiconductors form a triangular moiré lattice with high-symmetry sites *MM, MX* and *XM* (*M* = Mo, W; *X* = S, Se, Te). (b) Triangular lattice moiré potential for heterobilayers and homobilayers with K/K′-valley states. (c) Schematic moiré band structure in the first mBZ (mini-Brillouin zone) for heterobilayers with type-II band alignment. *E*_g1_ and *E*_g2_ are the bandgaps of the first and second TMD layers, respectively. (d) Topmost moiré valence bands for the K-valley state (blue solid line) and K′-valley state (orange dashed line) of AA-stacked twisted bilayer WSe_2_ under a perpendicular electric field *E* = 0 mV/nm (top panel) and 100 mV/nm (bottom panel). Arrows mark the van Hove singularities (vHS). (e) Honeycomb lattice moiré potential for twisted bilayer and multilayer TMDs with ${\mathrm{\Gamma }}$-valley states. (f) Topmost moiré valence bands for the ${\mathrm{\Gamma }}$-valley states in small-angle twisted double bilayer WSe_2_. The first set of moiré bands (thick lines) is graphene-like. Red dashed line: Fermi level at the Dirac point. (g) Schematic phase diagram of the triangular lattice moiré Hubbard model as a function of lattice filling factor *v* (near 1) and coupling. The dimensionless coupling constant $\frac{{U/W}}{{1 + U/W}}$ ∼ 1, 1/2 and 0 correspond to the strong, intermediate and weak coupling limits, respectively. The phase diagram is constructed based on data from both moiré heterobilayers and AA-stacked twisted bilayer WSe_2_. Experimental data are absent in the striped shaded region. FM, ferromagnetism; AF, antiferromagnetism; orange solid line, Mott/AF insulator; orange-shaded region, AF metal or doped Mott insulator; blue-shaded region, superconductor; dashed line, boundary for the strange metal (SM) phase. (a) and (c) are adapted from Mak and Shan [7]; (b) and (d–f) are from Wu *et al.* [10], Xia *et al.*  [37], Angeli and MacDonald [41] and Ma *et al.*  [43], respectively.

Monolayer TMDs (*MX*_2_, *M* = Mo, W; *X* = S, Se, Te) with a triangular lattice are direct bandgap semiconductors whose band edges reside at the K and K′ points of the Brillouin zone. Angle-aligned TMD heterobilayers, which form a triangular moiré lattice of 5–10 nm in period due to lattice mismatch between the two TMD monolayers, possess the simplest moiré band structure [7] (Fig. 1a–c). Lattice relaxations on the moiré length scale [8], which give rise to a periodically modulated strain and accordingly energy landscape for electrons, are the dominant mechanism for forming the moiré potential and moiré bands. The topmost moiré valence bands are nearly localized to one of the TMD layers because of the large valence band offset in typical TMD heterobilayers (Fig. 1c). The strong Ising spin-orbit splitting for the valence bands further reduces the spin-valley degeneracy to two [9]. In the limit of negligible inter-moiré-band mixing by Coulomb repulsions, the low-energy physics of the topmost moiré valence band can be mapped to a single-band triangular lattice Hubbard model [10].

The Hubbard model physics was demonstrated in experiments performed on angle-aligned TMD heterobilayers, such as WSe_2_/WS_2_, MoSe_2_/WS_2_, MoTe_2_/WSe_2_ etc. Because the moiré potential is typically strong, the Hubbard physics was realized mainly in the strong-coupling limit *U*/*W* > 1 (where W = 9*t* is the moiré bandwidth). The emergence of Mott insulators and local magnetic moments was observed at half-band-filling or full-lattice-filling denoted by lattice filling factor *v* = 1 [11,12]. The strong-coupling limit also provides a unique avenue to study the problem of kinetic magnetism (i.e. magnetism driven by the kinetic hopping term *t*) in doped Mott insulators [13,14]. Because of geometric and kinetic frustrations in a triangular lattice, both kinetic (or Nagaoka) ferromagnetism for *v* > 1 and kinetic antiferromagnetism for *v* < 1 were reported [15,16] (Fig. 1g); spin polarons (a bound state of a doped hole and a spin flip [17–19]) were observed in the latter case [16]. In addition to the onsite *U* term, the importance of the long-range Coulomb repulsions was also studied. A series of Wigner–Mott insulators [11,20] and stripe phases [21] was reported at fractional lattice fillings. To date, superconductivity has not yet been observed in angle-aligned TMD heterobilayers, likely due to the insufficient electronic kinetic energy in the strong-coupling limit [1].

## AA-STACKED TWISTED HOMOBILAYERS

AA-stacked twisted homobilayers (i.e. homobilayers with a small relative twist angle $\theta $) have recently attracted significant interest (in bulk TMD crystals with 2H structure, layers are aligned at 60° relative to each other). Here the periodically modulated interlayer hybridization on the moiré length scale contributes substantially to the moiré potential [22,23], although the lattice relaxation effects are still important, especially in the small twist angle limit [24,25]. Depending on the details of the moiré potential, the topmost moiré valence bands can be either topologically trivial or non-trivial [22,23,26]. In the topologically non-trivial case, both quantum spin Hall insulators and Chern insulators, as well as their fractionalized versions, were reported [27]. In the topologically trivial case, the bands can be mapped to a single-band triangular lattice Hubbard Hamiltonian [28–31] (again, in the limit of negligible inter-moiré-band mixing by Coulomb repulsions). Compared to the heterobilayer case, the moiré bandwidth *W* of twisted homobilayers is widely tunable through the twist angle; an electric field perpendicular to the sample plane can further tune *W* and introduce an emergent spin-orbit term in the Hamiltonian and spin-orbit splitting in the moiré bands [30] (Fig. 1d). The platform provides an opportunity to explore the Hubbard model physics with a continuously tunable *U*/*W* ratio, where coarse tuning is achieved through the twist angle [7] (U ∝ *θ*, W ∝ *θ*^2^ and *U*/*W* ∝ *θ*^−1^) and fine tuning, through the electric field.

Much progress has been made in this direction in the past few years with most studies featuring twisted bilayer WSe_2_. Specifically, a correlated insulator was reported at half-band-filling (*v* = 1) over a range of twist angle [32]. An electric-field-induced metal–insulator transition was demonstrated at *v* = 1 [33] (also in angle-aligned MoTe_2_/WSe_2_ heterobilayers [34]), supporting a bandwidth-tuned Mott transition [3]. With improved sample and device quality, robust superconductivity was recently reported near *v* = 1 [35,36]. Detailed twist-angle dependence studies [37,38] further showed that the superconducting state emerges right next to the bandwidth-tuned Mott transition. Near twist angle 3.5° (*U* ≥ *W*), a continuous superconductor-Mott insulator transition was observed near zero electric field at *v* = 1 [35]; here the Mott insulator is possibly non-magnetic [39,40]. Near 5° (*U* ≤ *W*), the superconducting state was observed right next to an antiferromagnetic metal near *v* = 1 at finite electric fields [36], where the van Hove singularity of the moiré band is moved by the electric field to the Fermi level, producing a nested Fermi surface [30]. At intermediate twist angles (*θ* = 4.6°), where U ∼ W and the correlated insulator at *v* = 1 barely survives, a phase diagram reminiscent of the iconic phase diagram of cuprate high-temperature superconductors was reported [37]. These results (Fig. 1g) strongly suggest that antiferromagnetism and/or Mott physics, rather than pure electron–phonon coupling, are the root cause of the observed superconductivity.

## Γ VALLEY MOIRÉ BANDS FOR THE HONEYCOMB LATTICE HUBBARD MODEL

Our discussions so far have focused on the K/K′-valley states of the valence band in TMDs. In AA-stacked twisted bilayer MS_2_ or multilayer *M*Se_2_ (*M* = Mo, W), the ${\mathrm{\Gamma }}$-valley states of the valence band are above the K/K′-valley states because the ${\mathrm{\Gamma }}$-valley states allow for substantially stronger interlayer hybridization. The ${\mathrm{\Gamma }}$-valley moiré bands are qualitatively different from their K/K′-valley counterparts [41,42]. A perpendicular electric field has weak effects on the moiré bands. Symmetry analysis shows that the moiré potential has a 6-fold symmetry [41,42] (Fig. 1e). Together with the negligible spin-orbit splitting for the ${\mathrm{\Gamma }}$-valley states, a topmost moiré valence band mimicking the iconic graphene band structure emerges (Fig. 1f), realizing a platform to explore Hubbard model physics in a bipartite honeycomb lattice (versus that in a non-bipartite triangular lattice for K/K′-valley states).

Recent experiments based on magnetoresistance and Hall effect measurements demonstrated the emergence of graphene-like flat bands in twisted double bilayer WSe_2_ with *θ* ≥ 2.7° [43]. The presence of a Dirac point, supporting massless fermions, is confirmed by the observation of a 4-fold degenerate zero-energy Landau level and a density-dependent cyclotron mass. In smaller twist angle samples (θ ≤ 2.7°), where the bands are flatter or equivalently the *U*/*W* ratio is larger, the Dirac point is spontaneously gapped out by Coulomb interactions; a bandwidth-tuned relativistic Mott transition from a semimetal to a Mott insulator was reported. Furthermore, a sufficiently large electric field can bring the K/K′-valley states to the Fermi level and introduce the mixed K/K′ valley and ${\mathrm{\Gamma }}$-valley states [43,44]; a valley-dependent charge-transfer insulator was reported in this regime [44].

## OUTLOOK

Semiconductor moiré materials provide a highly tunable platform to study the Hubbard model physics all the way from the large *U*/*W* limit, where electrons are particle-like, to the small *U*/*W* limit, where electrons are wave-like. In the large *U*/*W* limit, the system is ideal for exploring frustrated magnetism and kinetic magnetism in the vicinity of Mott and Wigner–Mott insulators. With reduced *U*/*W* ratio, especially for intermediate *U*/*W*, the system could help to shed new light on the physics of high-temperature superconductivity. Specifically, the gap symmetry of the observed superconducting state [31,45–58], the presence of a pseudogap phase [2] and/or other intertwined orders [4], and the nature of the observed strange metal phase [37] are outstanding questions to be addressed by future studies. The ability to tune the moiré bandwidth *W* continuously, especially by electric field in twisted homobilayers, also provides a unique opportunity to study the bandwidth-tuned phase transitions, such as the esoteric physics near the Wigner–Mott transitions [59] and the Gross–Neveu criticality and possible unconventional superconductivity near the relativistic Mott transition [60–64]. Last, in addition to the triangular and honeycomb lattice Hubbard models, a recent study proposed the realization of the square lattice Hubbard model in 90°-twisted Ge*X* or Sn*X* (*X* = S, Se) bilayers [65]. Exploring the latter could reveal new insight into cuprate superconductors, which are defined by 2D copper oxide square lattices.

Semiconductor moiré materials continue to push the frontiers of the study of electronic correlations, including those described by the Hubbard model. They provide many attractive features for simulation, complementing the more established platforms such as the cold atom systems. Particularly, heat extraction via the phonon bath is efficient and allows the moiré systems to be studied over a wide range of temperature, including ready access to the low temperature limit of pure quantum phase transitions. The gating procedures allow the phase diagram of model Hamiltonians to be explored over a wider range of density than is achievable in cold atom systems. It is also easier in principle to scale up the system size and to introduce long-range and spin–orbit interactions. However, significant challenges remain for simulation based on semiconductor moiré materials. New theoretical understanding and experimental methods are required to realize a highly controlled potential landscape for the electrons and to initiate, protect and measure their quantum many-body states.

## Notes

***Conflict of inerest statement.*** The authors declare no competing interests.

## References

[1] Arovas DP, Berg E, Kivelson SA et al. Annu Rev Condens Matter Phys 2022; 13: 239–74. 10.1146/annurev-conmatphys-031620-102024

[2] Lee PA, Nagaosa N, Wen X-G. Rev Mod Phys 2006; 78: 17–85. 10.1103/RevModPhys.78.17

[3] Imada M, Fujimori A, Tokura Y. Rev Mod Phys 1998; 70: 1039–263. 10.1103/RevModPhys.70.1039

[4] Fradkin E, Kivelson SA, Tranquada JM. Rev Mod Phys 2015; 87: 457–82. 10.1103/RevModPhys.87.457

[5] Quintanilla J, Hooley C. Phys World 2009; 22: 32–7. 10.1088/2058-7058/22/06/38

[6] Gross C, Bloch I. Science 2017; 357: 995–1001. 10.1126/science.aal3837 28883070

[7] Mak KF, Shan J. Nat Nanotechnol 2022; 17: 686–95. 10.1038/s41565-022-01165-6 35836003

[8] Li H, Li S, Naik MH et al. Nat Mater 2021; 20: 945–50. 10.1038/s41563-021-00923-6 33558718

[9] Xiao D, Liu G-B, Feng W et al. Phys Rev Lett 2012; 108: 196802. 10.1103/PhysRevLett.108.196802 23003071

[10] Wu F, Lovorn T, Tutuc E et al. Phys Rev Lett 2018; 121: 026402. 10.1103/PhysRevLett.121.026402 30085734

[11] Regan EC, Wang D, Jin C et al. Nature 2020; 579: 359–63. 10.1038/s41586-020-2092-4 32188951

[12] Tang Y, Li L, Li T et al. Nature 2020; 579: 353–8. 10.1038/s41586-020-2085-3 32188950

[13] Nagaoka Y . Phys Rev 1966; 147: 392–405. 10.1103/PhysRev.147.392

[14] Haerter JO, Shastry BS. Phys Rev Lett 2005; 95: 087202. 10.1103/PhysRevLett.95.087202 16196895

[15] Ciorciaro L, Smoleński T, Morera I et al. Nature 2023; 623: 509–13. 10.1038/s41586-023-06633-0 37968525 PMC10651480

[16] Tao Z, Zhao W, Shen B et al. Nat Phys 2024; 20: 783–7. 10.1038/s41567-024-02434-y

[17] Zhang S-S, Zhu W, Batista CD. Phys Rev B 2018; 97: 140507. 10.1103/PhysRevB.97.140507

[18] Davydova M, Zhang Y, Fu L. Phys Rev B 2023; 107: 224420. 10.1103/PhysRevB.107.224420

[19] Morera I, Kanász-Nagy M, Smolenski T et al. Phys Rev Res 2023; 5: L022048. 10.1103/PhysRevResearch.5.L022048

[20] Xu Y, Liu S, Rhodes DA et al. Nature 2020; 587: 214–8. 10.1038/s41586-020-2868-6 33177668

[21] Jin C, Tao Z, Li T et al. Nat Mater 2021; 20: 940–4. 10.1038/s41563-021-00959-8 33767398

[22] Wu F, Lovorn T, Tutuc E et al. Phys Rev Lett 2019; 122: 086402. 10.1103/PhysRevLett.122.086402 30932597

[23] Devakul T, Crépel V, Zhang Y et al. Nat Commun 2021; 12: 6730. 10.1038/s41467-021-27042-9 34795273 PMC8602625

[24] Zhang X-W, Wang C, Liu X et al. Nat Commun 2024; 15: 4223. 10.1038/s41467-024-48511-x 38762554 PMC11102499

[25] Jia Y, Yu J, Liu J et al. Phys Rev B 2024; 109: 205121. 10.1103/PhysRevB.109.205121

[26] Zhang F, Morales-Durán N, Li Y et al. Nat Phys 2025; 21: 1217–23.

[27] Bernevig BA, Fu L, Ju L et al. Nat Phys 2025; 21: 1702–13. 10.1038/s41567-025-03072-8

[28] Pan H, Wu F, Das Sarma S. Phys Rev Res 2020; 2: 033087. 10.1103/PhysRevResearch.2.033087

[29] Bi Z, Fu L. Nat Commun 2021; 12: 642. 10.1038/s41467-020-20802-z 33510138 PMC7843647

[30] Zang J, Wang J, Cano J et al. Phys Rev B 2021; 104: 075150. 10.1103/PhysRevB.104.075150

[31] Chubukov AV, Varma CM. Phys Rev B 2025; 111: 014507. 10.1103/PhysRevB.111.014507

[32] Wang L, Shih E-M, Ghiotto A et al. Nat Mater 2020; 19: 861–6. 10.1038/s41563-020-0708-6 32572205

[33] Ghiotto A, Shih E-M, Pereira GSSG et al. Nature 2021; 597: 345–9. 10.1038/s41586-021-03815-6 34526705

[34] Li T, Jiang S, Li L et al. Nature 2021; 597: 350–4. 10.1038/s41586-021-03853-0 34526709

[35] Xia Y, Han Z, Watanabe K et al. Nature 2025; 637: 833–8. 10.1038/s41586-024-08116-2 39478226

[36] Guo Y, Pack J, Swann J et al. Nature 2025; 637: 839–45. 10.1038/s41586-024-08381-1 39843588

[37] Xia Y, Han Z, Zhu J et al. Nature 2026; doi: 10.1038/s41586-025-10049-3.

[38] Guo Y, Cenker J, Fischer A et al. arXiv: 2512.06265.

[39] Myerson-Jain N, Xu C. arXiv: 2406.12971.

[40] Kim S, Mendez-Valderrama JF, Wang X et al. Nat Commun 2025; 16: 1701. 10.1038/s41467-025-56816-8 39962050 PMC11832926

[41] Angeli M, MacDonald AH. Proc Natl Acad Sci USA 2021; 118: e2021826118. 10.1073/pnas.2021826118 33658375 PMC7958387

[42] Pan H, Kim E-A, Jian C-M. Phys Rev Res 2023; 5: 043173. 10.1103/PhysRevResearch.5.043173

[43] Ma L, Chaturvedi R, Nguyen PX et al. Nat Mater 2025; 24: 1935–41. 10.1038/s41563-025-02359-8 41062624

[44] Wei L, Li Q, Rehman MU et al. Nat Commun 2025; 16: 1185. 10.1038/s41467-025-56490-w 39885166 PMC11782666

[45] Bélanger M, Fournier J, Sénéchal D. Phys Rev B 2022; 106: 235135. 10.1103/PhysRevB.106.235135

[46] Zegrodnik M, Biborski A. Phys Rev B 2023; 108: 064506. 10.1103/PhysRevB.108.064506

[47] Klebl L, Fischer A, Classen L et al. Phys Rev Res 2023; 5: L012034. 10.1103/PhysRevResearch.5.L012034

[48] Christos M, Bonetti PM, Scheurer MS. Phys Rev Lett 2025; 135: 046503. 10.1103/7z4z-vlj8 40794036

[49] Tuo C, Li M-R, Wu Z et al. Nat Commun 2025; 16: 9525. 10.1038/s41467-025-64519-3 41152276 PMC12569151

[50] Akbar W, Biborski A, Rademaker L et al. Phys Rev B 2024; 110: 064516. 10.1103/PhysRevB.110.064516

[51] Xie F, Chen L, Sur S et al. Phys Rev Lett 2025; 134: 136503. 10.1103/PhysRevLett.134.136503 40250373

[52] Xie F, Li C, Cano J et al. arXiv: 2503.21769.

[53] Wu Y-M, Wu Z, Yao H. Phys Rev Lett 2023; 130: 126001. 10.1103/PhysRevLett.130.126001 37027848

[54] Guerci D, Kaplan D, Ingham J et al. arXiv: 2408.16075.

[55] Fischer A, Klebl L, Crépel V et al. arXiv: 2412.14296.

[56] Schrade C, Fu L. Phys Rev B 2024; 110: 035143. 10.1103/PhysRevB.110.035143

[57] Zhu J, Chou Y-Z, Xie M et al. Phys Rev B 2025; 111: L060501. 10.1103/PhysRevB.111.L060501

[58] Qin W, Qiu W-X, Wu F. Phys Rev Lett 2025; 135: 246002. 10.1103/kf2b-r9g5 41482288

[59] Musser S, Senthil T, Chowdhury D. Phys Rev B 2022; 106: 155145. 10.1103/PhysRevB.106.155145

[60] Herbut IF . Phys Rev Lett 2006; 97: 146401. 10.1103/PhysRevLett.97.146401 17155272

[61] Herbut IF, Juričić V, Vafek O. Phys Rev B 2009; 80: 075432. 10.1103/PhysRevB.80.075432 18352311

[62] Stangier VC, Sheehy DE, Schmalian J. arXiv: 2509. 25318.

[63] Stangier VC, Scheurer MS, Sheehy DE et al. arXiv: 2510.06313.

[64] Kim J . arXiv: 2510.18875.

[65] Xu Q, Fischer A, Tancogne-Dejean N et al. Phys Rev X 2025; 15: 041049.

